# Revisiting Bioresorbable Scaffolds in Light of the SCAAR Study

**DOI:** 10.1016/j.jscai.2025.104050

**Published:** 2025-11-11

**Authors:** Tirayut Veerasathian, Schawanya K. Rattanapitoon, Nav La, Nathkapach K. Rattanapitoon

**Affiliations:** Department of Surgery, Buriram Hospital, Buriram, Thailand; FMC Medical Center of Thailand, Nakhon Ratchasima, Thailand; Faculty of Pediatrics and Medicine, International University, Phnom Penh, Cambodia; FMC Medical Center of Thailand, Nakhon Ratchasima, Thailand

The long-term SCAAR analysis by Saidi-Seresht et al^1^ provides an important perspective on bioresorbable scaffolds (BRS). Although early scaffold thrombosis (∼3%) and restenosis (∼7%) were observed, myocardial infarction rates declined by approximately 25% beyond 3 years, highlighting the potential of vascular restoration and suggesting that the concept of “leaving nothing behind” may yet hold promise.

Age appears to modify this benefit. Patients aged ≥70 years experienced higher early risk without clear long-term advantage, likely reflecting more calcified vessels, impaired endothelial repair, and limited life expectancy. Younger patients, in contrast, may uniquely benefit from scaffold resorption and restored vasomotor function. This observation emphasizes that BRS outcomes are inherently patient- and time-dependent.

Emerging platforms offer a hopeful path forward. Magnesium-based scaffolds (DREAMS 3G) and sirolimus-eluting bioadaptors (BIODAPTOR-RCT) have demonstrated improved procedural safety and lower early adverse events compared with Absorb.^2^^,^^3^ If these devices maintain safety while enabling meaningful long-term vascular restoration, they may allow BRS to fulfill the promise originally envisioned.

Clinically, BRS should not be viewed as a one-size-fits-all alternative to drug-eluting stents. Thoughtful patient selection—avoiding elderly or high-risk anatomies and focusing on younger, lower-risk individuals—may allow us to realize the potential of this innovative approach while preserving safety.
